# ASAS-NANP symposium: mathematical modeling in animal nutrition: review of time series techniques and machine learning models for advancing livestock production

**DOI:** 10.1093/jas/skaf439

**Published:** 2025-12-18

**Authors:** Jesto Peter, Mo Li, Jameson Brennan, Hector Menendez, Hossein Moradi Rekabdarkolaee

**Affiliations:** Department of Business Analytics, Economics, and Information Systems, Bowling Green State University, Allen W. and Carol M. Schmidthorst College of Business Bowling Green, OH, 43403, United States; Department of Mathematics, University of Louisiana at Lafayette, Lafayette, LA 70504, United States; South Dakota State University West River Research and Extension Center 711 N. Creek Dr., Rapid City, South Dakota, United States; South Dakota State University West River Research and Extension Center 711 N. Creek Dr., Rapid City, South Dakota, United States; Department of Business Analytics, Economics, and Information Systems, Bowling Green State University, Allen W. and Carol M. Schmidthorst College of Business, Bowling Green, OH, 43403, United States

**Keywords:** ARIMAX, autocorrelation, deep learning, dynamic models, learning rate, linear models

## Abstract

Correlated data, including time series where observations depend on previous values, frequently occur in various livestock production studies. There are many statistical models and machine learning algorithms that have been developed to analyze this type of data. This paper provides a review of these models. Specifically, a detailed overview of the AutoRegressive Integrated Moving Average with eXogenous inputs (ARIMAX) model, Dynamic Models and Kalman Filtering (KF), Deep Neural Network (DNN), Recurrent Neural Network (RNN), and Transformer models are provided. This review focuses on steps to train different statistical and machine learning models and lists the advantages and limitations of each model.

## Teaser

Within agricultural systems, data collection often occurs over time. Essential to analyzing these data to understand trends and patterns over time and to forecast predictions into the future is to account for the temporal autocorrelation of the data.

## Introduction

A common data type within animal production systems is time series because animals are biological systems that have a definite start and end point relative to production goals, phase, and species, unlike other commodities that can be stored like engines or computer chips. Time series is an ordered sequence of observations where the ordering occurs over time (Shumway et al., 2000; Hamilton, 2020). In other words, a time series is based on observations that are collected sequentially in time. Examples of time series data are hourly wind speed, daily and monthly average air temperature, annual rainfall, and livestock full body weight collected over time. The objectives of time series analysis are: description, explanation, and prediction. Description of a time series is analogous to descriptive statistics in analyzing independent data and determining what trends and patterns a time series has by plotting or using more complex techniques. The most basic approach for data description is to graph the time series and look at: overall trends (e.g. increase, decrease, etc) and cyclic patterns (e.g. seasonal effects, etc). Explanation of a time series is to identify the underlying factors or causes that drive the observed patterns. This might involve looking for relationships between variables or determining if external events influence the time series. For instance, you could analyze how changes in precipitation patterns influence forage quality and quantity for grazing animals. Prediction of a time series analysis is widely used for forecasting future values based on historical data. Using models like AutoRegressive Integrated Moving Average (ARIMA), exponential smoothing, or machine learning techniques, the goal is to predict future values of the time series with some level of accuracy. A time series model with AutoRegressive (AR), Integrated (I) and Moving Average (MA) components is referred to as an ARIMA model. It operates under the assumption that future values of a time series are primarily determined by its past values, with differencing applied to address non-stationarity. However, in practical applications, relying solely on past values of the series may be overly simplistic, limiting the model’s ability to account for external influences. A time series is a combination of the trend, seasonality, and noise. Trend and seasonal variation are often referred to as large variation. Seasonality is a periodic pattern in the time series. One of the primary objectives of building a model for a time series is to be able to forecast the values for that series at future times. The assessment of the accuracy, precision, and bias of those forecasts are very important.

Traditional statistical methods, such as ARIMA and exponential smoothing, are widely used to model time-dependent data for prediction and analysis (Box et al., 2015). However, with the rise of Deep Neural Networks (DNN), new methods have emerged that can handle complex, non-linear relationships and large volumes of data. Deep learning approaches, especially recurrent neural networks (RNNs) and long short-term memory (LSTM) networks, have gained significant attention due to their ability to model sequential data and learn long-term dependencies in time series (Hu et al., 2018; Staudemeyer and Morris, 2019; Lindemann et al., 2021). These methods are useful when dealing with large datasets and intricate patterns that traditional statistical models may not fully capture, marking a shift toward more advanced and automated forecasting systems (Zhang et al., 1998). While statistical models remain important for simpler, well-understood data, DNN models offer more powerful tools for handling complex time series data in modern applications.

## AutoRegressive Integrated Moving Average with eXogenous Inputs (ARIMAX) Model

Time series forecasting is a fundamental task that involves using historical data to predict future values. The AutoRegressive Integrated Moving Average with eXogenous Inputs (ARIMAX) model is a parametric approach that is often used for forecasting when there exists a temporal correlation in the data (Shumway et al., 2000). The ARIMAX model contains four main components: AutoRegressive (AR), Integrated (I), Moving Average (MA), and the external (exogenous) variables that are believed to influence the response variable. The ARIMAX model adds external explanatory variables to improve the accuracy of forecasts by incorporating the influence of additional information outside of the time series itself.

The AR component represents the current value of the time series as a linear function of its past values, making it particularly useful when the series exhibits significant autocorrelation, where observations are correlated with their past values at different time lags. Mathematically, the AR part of the model is represented as:


$$y_{t}=\phi_{1}\;y_{t-1}\;+\phi_{2}\;y_{t-2}\;+\cdots \;+\phi_{p}\;y_{t-p}\;+\epsilon_{t},$$

where $y_{t}$ represents the current value at time t, $\left(\phi_{1},\;\phi_{2},\;\ldots ,\;\phi_{p}\right)$ are the parameters of the AR component, p is the order of the AR process, and $\epsilon_{t}$ is the error term at time t.

The Integrated (I) component addresses non-stationarity in a time series by applying the differencing operator. A time series is considered stationary if its statistical properties, such as mean and variance, remain constant over time (Shumway et al., 2000). When a series exhibits non-stationarity, differencing transforms it by computing the difference between consecutive observations, thereby stabilizing the mean and facilitating stationarity. The first-order differencing is given by


$$\nabla^{1}\;y_{t}=\;y_{t}\;-\;y_{t-1}.$$

If the series remains non-stationary after applying the first-order differencing operator ($\nabla^{1}$), higher-order differencing operators can be applied.

The Moving Average (MA) component models the relationship between the current value of the time series as a linear function of the past forecast errors. This helps capture the random shocks or noise that could influence the time series. The general form of the MA model is expressed as:


$$y_{t}\;=\mu \;+\theta_{1}\epsilon_{t-1}\;+\theta_{2}\epsilon_{t-2}\;+\cdots \;+\;\theta_{q}\epsilon_{t-q}\;+\epsilon_{t},$$

where μ is the mean of the time series, $\theta_{1},\;\theta_{2},\;\ldots ,\;\theta_{q}$ are the MA parameters, q is the order of the MA process, and $\epsilon_{t}$ is the error term at time t.

If the exogenous variables play a crucial role in shaping the trajectory of a time series, the ARIMAX model extends the ARIMA model and can incorporate these external variables to improve the model’s predictive accuracy. The ARIMAX(p, d, q) model equation is given as:


$$\nabla^{d}y_{t}\;=\sum_{i=1}^{p}\phi_{i}\;\nabla^{d}y_{t-i}+\sum_{j=1}^{q}\theta_{j}\epsilon_{t-j}+\sum_{k=1}^{r}\beta_{k}X_{k,t}+\epsilon_{t},$$

where $\nabla^{d}y_{t}$ represents the d^th^ order differenced dependent series at time t, $X_{1,t},\;\ldots ,\;X_{r,t}$ are the exogenous variables at time t with the associated coefficients $\beta_{1},\;\ldots ,\;\beta_{q},\;\phi_{1},\;\phi_{2},\;\ldots ,\;\phi_{p}$ are the AR coefficients, $\theta_{1},\;\theta_{2},\;\ldots ,\;\theta_{q}$ are the MA coefficients, and $\epsilon_{t}$ is the error term. The inclusion of exogenous variables allows the model to account for external influences on the dependent variable, such as marketing campaigns (Saputra and Yadav, 2024), economic indicators (Peter and Silvia, 2012), infectious diseases (Chadsuthi et al., 2015; Hossain et al., 2021) or weather conditions (Islam and Imteaz, 2021), which may significantly affect the underlying time series dynamics.

To implement an ARIMAX model, several critical steps are typically involved:

1. **Data Preprocessing:** Preparing the time series data is a crucial step and involves tasks such as outlier detection (Akouemo and Povinelli, 2014), missing value imputation (Akouemo and Povinelli, 2014; Nozari et al., 2023), and ensuring the correct alignment of exogenous variables with the dependent variable. Additionally, examining stationarity is essential when fitting an ARIMAX model. Through differencing at appropriate lags and orders, most time series can be transformed into a stationary form with a constant mean and variance over time, improving forecasting reliability. The differencing order is selected based on the minimum number of differencing needed to achieve stationarity. To assess stationarity, hypothesis testing methods like the Augmented Dickey-Fuller (ADF) test (Mushtaq, 2011) and the Kwiatkowski-Phillips-Schmidt-Shin (KPSS) test (Kwiatkowski et al., 1992) are frequently used.
2. **Model Selection:** Choosing the appropriate orders for the AR and MA components (determine the integer values of p and q) is a critical step to specify the internal structure of the time series. To guide this selection, the sample autocorrelation function (ACF) and partial autocorrelation function (PACF) are commonly used (Shumway et al., 2000), and the general behavior of the ACF and PACF plots for ARIMA model specification were discussed in Cryer and Chan (2008). Additionally, information criteria such as the Akaike Information Criterion (AIC), Bayesian Information Criterion (BIC), and Corrected Akaike Information Criterion (AICc) can further confirm the optimal model order by balancing goodness of fit with model complexity (Hurvich and Tsai, 1989; Cryer and Chan, 2008).
3. **Model Estimation:** With the determined model orders, p, d, and q, and the exogenous variables, we can obtain the model parameter estimates via various methods. Maximum Likelihood Estimation (MLE) is one of the most commonly used methods, as it maximizes the likelihood function and provides asymptotically efficient estimates, particularly useful in complex ARIMA and ARIMAX models with exogenous variables (Shumway et al., 2000). Least Squares Estimation, using the Yule-Walker equations, offers a computationally efficient method for AR models by relating autocorrelations to AR parameters (Shumway et al., 2000). While it is simpler and often used for AR models, it may not be as robust as MLE (Cryer and Chan, 2008). Conditional Sum of Squares (CSS) is another approach often applied in ARIMA and ARIMAX models, minimizing residual sums conditioned on past observations (Hurvich and Tsai, 1989). Kalman filtering (KF) is useful for real-time parameter estimation in dynamic time series models, such as ARIMAX models in state-space form (Durbin and Koopman, 2012). The choice of method depends on the model’s complexity, the extent of available data, and computational considerations.
4. **Model Validation:** Validation typically includes the residual analysis for model adequacy checks. Residual diagnostics assess autocorrelation, normality, and heteroscedasticity, which are important assumptions in time series modeling (Shumway et al., 2000). The Ljung-Box test (Ljung and Box, 1978) can be used to check for autocorrelation in the residuals, helping confirm whether the model captures the temporal dependencies in the data. Model validation also includes out-of-sample forecasting, where the model’s predictive performance is evaluated on unseen data. The time series is split into training and testing sets, and the cross-validation techniques, such as walk-forward validation (Tashman, 2000) and blocked time series validation (Bergmeir and Benítez, 2012), were used to ensure that the model not only fits the historical data well but also has good generalizability to future observations.
5. **Forecasting:** After fitting the ARIMAX model, it can be used to generate forecasts by including future values of the exogenous variables. These forecasts include both point predictions and prediction intervals, which estimate a range of potential outcomes with a specified confidence level. To evaluate the model’s forecasting accuracy, common metrics like Mean Absolute Error (MAE), Root Mean Squared Error (RMSE), and Mean Absolute Percentage Error (MAPE) are employed, providing insight into the magnitude of prediction errors and the model’s overall performance (Hyndman and Athanasopoulos, 2018).

The ARIMAX model is a versatile tool for time series forecasting that not only accounts for past data patterns but also incorporates external factors. By combining autoregression, differencing, and moving averages, it can capture both the natural flow from the data itself and the impact of outside influences. This makes it especially valuable in areas like economics, finance, and retail, where understanding both historical trends and external variables is key to making accurate predictions.

## Linear Dynamic Models

A linear dynamic system can be described by the following state-space equations (Kalman, 1960):


(1) x(t+1)= Ax(t)+ Bu(t)+ w(t)

(2) y(t)= Cx(t)+ Du(t)+ν(t)

where x(t) is the state vector at time t, u(t) is the control input at time t, y(t) is the measurement at time t, A, B, C, D, are system matrices, w(t) and ν(t) represent process and measurement noise, respectively.

## Kalman Filtering

The Kalman filter is a recursive algorithm to estimate the state of a system given noisy measurements (Kalman, 1960; Grewal and Andrews, 2014). The prediction equations are:


(3) $$\hat{x}(t+1|t)=\;A\hat{x}(t)+\;Bu(t)$$

(4) $$P(t+1|t)=\;AP(t)A^{T}+Q$$

where $\hat{x}(t+1|t)$ is the predicted state estimate, P(t+1|t) is the predicted covariance, Q is the process noise covariance. The updated equations are:


(5) $$K(t+1)=\;P(t+1|t)C^{T}\;{\left(C\;P(t+1|t)C^{T}+R\right)}^{-1}$$

(6) $$\hat{x}(t+1|t+1)=\;\hat{x}(t+1|t)+\;K(t+1)[y(t+1)-\;C\hat{x}(t+1|t)]$$

(7) P(t+1|t+1)= (I - K(t+1)C)P(t+1|t)

where K(t+1) is the Kalman gain, R is the measurement noise covariance, $\hat{x}(t+1|t+1)$ is the updated state estimate, and P(t+1|t+1) is the updated covariance. The Kalman filter provides a powerful approach for estimating the state of a system, with applications in many fields such as robotics, economics, and aerospace (Grewal and Andrews, 2014).

A common assumption is that the function *f* depends on several (unknown) *parameters* $\beta_{1},\ldots ,\;\beta_{p}$, i.e.,


$$f(t)\;=f(t;\beta_{1},\;\ldots ,\;\beta_{p}).$$

However, the *type* of the function f is known. The parameters $\beta_{1},\;\ldots ,\;\beta_{p}$ are needed to be estimated from the data. A common approach is a least squares estimate ${\hat{\beta }}_{1},\;\ldots ,\;{\hat{\beta }}_{p}$ satisfying


$$\underset{\beta_{1},\ldots ,\;\beta_{p}}{\min}\sum {\left(y_{t}-f(t;\beta_{1},\ldots ,\beta_{p})\right)}^{2}.$$

The ${\hat{y}}_{t}=f(t;\beta_{1},\;\ldots ,\;\beta_{p})$ provide an estimation of the trend function. The residuals, $y_{t}-{\hat{y}}_{t}$, provide an estimate of $\epsilon_{t}$.

## State Estimation

State estimation with a Kalman filter is a technique that uses a linear model to estimate the state of a system using noisy measurements. The Kalman filter is also known as linear quadratic estimation (LQE).

Linear Filtering is a method that deals with a trend. Specifically, let $a_{i},\;i=-q,-q+1,\ldots \;s$ be arbitrary real numbers, where q,s≥0, q+s+1≤n. The linear transformation


$$y^{*}(t):=\sum_{i=-q}^{s}a_{i}\;y(t-i),\;t=s+1,\ldots ,\;n-q,$$

converts one time series {y(t)} into another, $\left\{y^{*}(t)\right\}$. This transformation is called a *linear filter* with *weights* $\left\{a_{i}\right\}$. The y(t) are called *input* and the $y^{*}(t)$ are called *output*. Exponential smoothing is another popular way of smoothing or filtering a time series. In this case


$$Sm(y_{t})=y_{t}^{*}\;=\sum_{j=0}^{\infty }\alpha {\left(1-\alpha \right)}^{j}{\;}_{t-j},$$

where 0<α<1is a constant. Here we note that the weights $a_{j}=\alpha {\left(1-\alpha \right)}^{j}$, that decreases geometrically with j.

Suppose $y_{t}$ be the response variable at time t=1,…, T, is modeled as


(8) y(t)=Λ(t)X(t)+ϵ(t)

and the evolution model for time t=1,…, T is,


(9) X(t)=Γ(t−1)X(t−1)+ζ(t)

The vector X(t) is the independent variables also called the state of the system, ΛΛ(t) is the measurement model matrix, ΓΓ(t) is the state transition matrix of the dynamic model, ϵ(t) is the process noise and ζ(t) is the measurement noise. The KF provides a closed-form solution to linear Gaussian filtering. Linearity of the system and measurement models:


$$y_{t}\;=\;F_{t}\;y_{t-1}\;+\;B_{t}u_{t}+\epsilon_{t},$$

where $F_{t}$ is the state transition model, $B_{t}$ is the control-input model applied to the control vector $u_{t}$. Noise within the system and measurements are Gaussian. The extended Kalman filter (EKF) uses Taylor series expansion to model non-linear and non-Gaussian measurements.

## Deep Learning and Machine Learning Algorithms for Time Series Data

### Machine learning and deep learning

Machine Learning (ML) is a subset of artificial intelligence (AI) that allows systems to automatically learn and improve from experience without being explicitly programmed (Bini, 2018). Instead of following pre-defined instructions, ML algorithms enable machines to analyze data, recognize patterns, and make predictions based on that data. The core concept is that systems can learn from past data and improve their performance as they process more data over time (Jordan and Mitchell, 2015).

Machine learning can be categorized into three main types (­Ayodele, 2010). In supervised learning, the algorithm learns from labeled data, where input-output pairs are provided, and the goal is to map inputs to the correct outputs, typically used for classification and regression tasks. In unsupervised learning, the algorithm works with unlabeled data, aiming to discover hidden patterns or structures, such as clustering similar items or reducing the dimensionality of data. Both supervised and unsupervised learning approaches have been used, for example, to categorized livestock grazing behavior within rangeland systems (Brennan et al., 2021; Liu et al., 2024). Reinforcement Learning involves an agent interacting with its environment, receiving feedback in the form of rewards or penalties, and learning to maximize the cumulative reward over time through trial and error.

**Figure 1.**
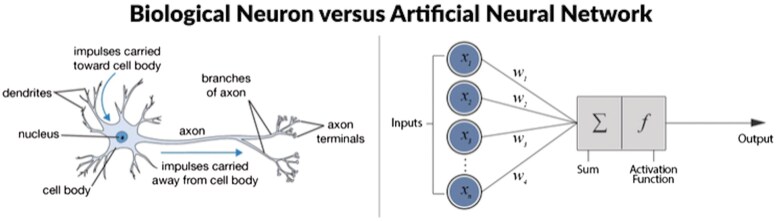
Biological neuron vs artificial neural network. Source: Willems (2018).

Deep Learning (DL) is a subset of machine learning that focuses on algorithms inspired by the structure and function of the human brain. These algorithms are known as neural networks. Figure 1 shows the structure of a biological neuron vs. an Artificial Neural Network. Deep learning involves multiple layers of these neural networks (hence the term “deep”) to learn from large datasets and automatically extract features for prediction (Taye, 2023). This structure is what makes deep learning capable of handling complex tasks, such as image and speech recognition, language translation, and autonomous driving (Sarker, 2021).

While traditional machine learning methods may require manual feature extraction from raw data, deep learning automates this process (Patil et al., 2024), making it more suitable for tasks involving unstructured data like images, audio, and text.

### Key differences between machine learning and deep learning

Although machine learning and deep learning are closely related, there are several key differences between the two approaches. Machine learning works well with smaller datasets and requires manual feature extraction, while deep learning excels with large datasets and can automatically learn features from raw data. Deep learning models are more complex and computationally expensive, often requiring Graphics Processing Units (GPUs) or Tensor Processing Units (TPUs) for training. Machine Learning models are typically easier to interpret, while deep learning models are often considered “black-box” models due to their complexity. Machine Learning is best for simpler tasks, while deep learning is ideal for more complex tasks such as image and speech recognition (Janiesch et al., 2021).

### Historical context: the rise of deep learning

The evolution of neural networks and deep learning can be traced back to the 1950s and 1960s. In 1957, Frank Rosenblatt introduced the Perceptron, the first simple neural network, which laid the groundwork for modern neural networks. This early model was a single-layer neural network capable of performing binary classification tasks. In the 1980s, algorithms for adjusting neural network weights were developed, which enabled deeper networks with more layers. However, the lack of interpretability and the difficulty of training deep networks led to a decline in the popularity of neural networks in favor of support vector machines and other methods (Macukow, 2016).

The resurgence of deep learning in the 2000s was largely due to advancements in GPU technology. Graphics Processing Units, initially developed for gaming, have thousands of cores capable of performing parallel computations, making them ideal for the intense computational demands of deep learning (Dally et al., 2021). With the rise of large datasets and powerful computing resources, deep learning made a dramatic comeback and is now a central component of modern AI systems.

### How neural networks work

Neural networks are the backbone of deep learning. They consist of three main types of layers:

1. **Input Layer:** This is where the input data is fed into the neural network. The input layer consists of neurons that represent the features of the data (e.g., pixel values for images, word embedding for text).
2. **Hidden Layers:** These layers are where the actual computation happens. Data passes through multiple hidden layers, where weights and activation functions are applied to modify the inputs. Each layer transforms the data to extract higher-level features.
3. **Output Layer:** This layer generates the final output or prediction. For classification tasks, this might be a probability distribution over classes, while for regression tasks, it could be a continuous value.

Training a neural network involves adjusting the weights of the network using an optimization algorithm such as gradient descent. During training, the network compares its predictions with the actual values (ground truth) and computes the error. The error is then back propagated through the network to adjust the weights, minimizing the error over time (Islam et al., 2019).

### Activation functions

An activation function is a mathematical operation applied to the output of each neuron in a neural network. Its primary purpose is to introduce non-linearity into the network, enabling the model to learn and represent complex patterns in data. Without activation functions, a neural network would essentially become a linear model, even if multiple layers are used. This linearity would significantly limit the model’s ability to approximate real-world data and solve complex problems, such as classification or regression tasks, effectively (Sharma et al., 2020).

Activation functions are crucial in enabling neural networks to solve tasks that involve non-linear relationships. They determine whether a neuron should be activated or not, essentially deciding if the information should be passed to the next layer of the network (Nwankpa et al., 2018). Without them, the network would be unable to learn intricate patterns in data, as all the transformations performed by the layers would simply be linear. Common activation functions include: Sigmoid, Rectified Linear Unit (ReLU), Tangent Hyperbolic (Tanh).

## The Evolution of Neural Networks: From Perceptron to Transformers

### The perceptron and the multilayer perceptron

When neural networks expand beyond a single layer, they become Multilayer Perceptrons (MLPs). Multilayer perceptrons are composed of an input layer, one or more hidden layers, and an output layer. The introduction of multiple layers allows MLPs to model more complex relationships and represent non-linear decision boundaries. The MLP architecture is capable of learning and mapping complex functions, and it is also trained using backpropagation to minimize the error through each layer by adjusting the weights (Noriega, 2005).

### Recurrent neural networks

Recurrent Neural Networks are an extension of traditional neural networks designed to handle sequential data, making them especially useful for tasks such as time-series analysis, speech recognition, and language modeling. Unlike feedforward neural networks, RNNs have connections that allow information to persist across time steps, creating a “memory” of previous inputs in the sequence (Schmidt, 2019).

### RNN architecture

The key feature of RNNs is the recurrence relation that enables information from previous time steps to influence the current output (Mienye et al., 2024). The basic structure of an RNN includes an input layer, a hidden state that is updated at each time step, and an output layer. The hidden state is computed as a function of both the previous hidden state and the current input.

The recurrence relation for an RNN at time step t is given by:


$$h_{t}\;=\sigma (W_{h}h_{t-1}\;+\;W_{x}\;x_{t}+b),$$

where $h_{t}$ is the hidden state at time step t, $h_{t-1}$ is the hidden state from the previous time step, $x_{t}$ is the input at time step t, $W_{h}$ and $W_{x}$ are weight matrices for the hidden state and the input, b is the bias term, σ is an activation function (commonly tanh or ReLU). The output at each time step is computed as:


$$y_{t}=\;g(W_{y}\;h_{t}\;+\;b_{y}),$$

where $y_{t}$ is the output at time step t, $W_{y}$ is the weight matrix for the output, $b_{y}$ is the output bias and g is the activation function for the output layer.

### Challenges with RNNs

Despite their ability to handle sequential data, RNNs suffer from two major challenges (Mienye et al., 2024): The first challenge of RNNs is known as the Vanishing Gradient Problem. When training RNNs with backpropagation through time (BPTT), gradients can become extremely small and “vanish” as they are propagated back through many time steps. This makes it difficult for RNNs to learn long-range dependencies. The second issue with RNNs is the Exploding Gradients problem. In contrast to vanishing gradients, gradients can also explode, leading to unstable updates and causing the model to fail during training. To address these issues, more advanced models like LSTM networks were developed.

### Long short-term memory networks

Long short-term memory networks are a type of RNN designed to overcome the vanishing gradient problem and improve the ability of the network to learn long-term dependencies in sequential data. Long short-term memory introduce a special architecture that includes memory cells and gating mechanisms to control the flow of information (Staudemeyer and Morris, 2019).

### LSTM architecture

An LSTM unit consists of three main gates: the input gate, the forget gate, and the output gate (Hu et al., 2018). These gates control how information is updated and passed along through the network. Each LSTM unit also has a memory cell that stores long-term information, allowing the network to retain relevant information over long sequences.

The key equations for an LSTM unit at time step t are as follows:

1. **Forget Gate:** Determines what proportion of the previous memory $C_{t-1}$ to forget:
  $$f_{t}=\sigma (W_{f}\;h_{t-1}\;+\;W_{f}\;x_{t}\;+\;b_{f}).$$
2. **Input Gate:** Decides what new information to store in the memory:
  $$i_{t}=\sigma (W_{i}\;h_{t-1}\;+\;W_{i}\;x_{t}\;+\;b_{i}).$$
3. **Memory Update:** Updates the memory cell based on the forget and input gates:
  $$C_{t}=\;f_{t}*C_{t-1}+i_{t}\;*{\tilde{C}}_{t}.$$
4. **Output Gate:** Determines what part of the memory cell to output as the hidden state:
  $$o_{t}=\sigma (W_{o}h_{t-1}+W_{o}x_{t}+b_{o}),$$
  $$h_{t}\;=\;o_{t}\;*\;\tanh(C_{t}),$$

where $x_{t}$ is the input data, σ is the sigmoid activation function, tanh is the hyperbolic tangent activation function, $W_{f},\;W_{i},\;W_{o}$ are weight matrices for each gate, $b_{f},\;b_{i},\;b_{o}$ are bias terms. The output $h_{t}$ and the updated memory $C_{t}$ are passed to the next time step.

### Advantages of LSTMs

Long short-term memory are particularly effective in modeling long-term dependencies due to their ability to remember relevant information over many time steps, preventing the vanishing gradient problem that RNNs suffer from (Song et al., 2020). They have been widely used in applications such as speech recognition, machine translation, and time-series forecasting (Ren, 2020; Yang et al., 2020; Lindemann et al., 2021).

### Drawbacks of RNNs and LSTMs with time-series datasets

Although LSTMs have significantly improved upon the capabilities of basic RNNs, they still face challenges when working with long time-series datasets. One of the main issues is the inherent sequential nature of RNNs and LSTMs, which makes them computationally expensive to train, especially for very long sequences. Additionally, LSTMs still struggle with modeling dependencies over very long sequences, and their performance can degrade when dealing with noisy or highly variable data (Kandadi and Shankarlingam, 2025). To address the limitations of RNNs and LSTMs, particularly in handling long-term dependencies in sequential data, the Transformer model was introduced in the paper “Attention is All You Need” by Vaswani et al. (2017).

### Transformers

Transformers have fundamentally altered the landscape of deep learning, particularly in tasks involving sequential data (Kamatala et al., 2025). Initially introduced by Vaswani et al. (2017), transformers have become the foundation for numerous state-of-the-art models, especially in natural language processing (NLP; Kalyan et al., 2021). The core components of the transformer model include the encoder and decoder, each comprising multiple layers of self-attention and feed-forward networks (FFNs). At the heart of the transformer model is the concept of parallelization and attention, enabling it to model dependencies across long sequences more efficiently than traditional methods like RNNs or LSTMs (Zhang et al., 2023). This makes transformers particularly effective in fields such as time series forecasting and agricultural data analysis, where sequential dependencies play a crucial role (Zhu et al., 2023; Bilel, 2024).

### Time series modeling with transformers

Traditional methods like ARIMA and exponential smoothing have been widely used for time series forecasting, but their limitations in handling nonlinear patterns and long-term dependencies have led to the adoption of deep learning approaches such as RNNs, LSTMs, and, more recently, transformers (Mujiyanto et al., 2024). The self-attention mechanism in the transformer allows the model to focus on the most relevant parts of the input sequence when making predictions (Choi and Lee 2023). This is particularly useful in time series data, where patterns from distant time steps might be highly relevant for future predictions (Peter et al., 2025).

For time series forecasting, the encoder processes the input sequence, and the decoder generates the forecasted values. In some configurations, only the encoder is used for autoregressive forecasting, where predictions are made one time step at a time, and previous predictions are used as inputs for subsequent steps (Ahmed et al., 2023). The attention mechanism is central to how transformers process the input data.

### Self-attention mechanism

The self-attention mechanism calculates a weight for each element of the input sequence, which determines how much attention should be given to each element when predicting the output. Mathematically, the self-attention mechanism can be described as (Vaswani et al., 2017):


$$\mathrm{Attention}(Q,\;K,\;V)\;=\;\mathrm{softmax}(\frac{QK^{T}}{\sqrt{d_{k}}})V,$$

where Q is the query matrix, K is the key matrix, V is the value matrix, and $d_{k}$ is the dimension of the key vectors. The key idea behind self-attention is to compute the dot product between the query Q and key K matrices to obtain the attention scores. These scores are then normalized using the softmax function, and finally, a weighted sum of the value matrix V is computed, which represents the output of the attention mechanism. This allows each position in the sequence to “attend” to other positions, creating a contextualized representation of the input sequence that captures both local and global dependencies.

### Feedforward networks and activation functions

After the self-attention mechanism, the transformer model passes the output through a FFN. The FFN consists of two fully connected layers, typically with a Rectified Linear Unit (ReLU) activation function in between.


$$\mathrm{FFN}(x)=\max(0,\;xW_{1}+\;b_{1})W_{2}+b_{2},$$

where *x* is the input vector, $W_{1}$ and $W_{2}$ are weight matrices for the first and second layers of the FFN, $b_{1}$ and $b_{2}$ are the bias terms. The ReLU activation function is commonly used because of its simplicity and effectiveness in mitigating the vanishing gradient problem (Szanda la, 2021). The FFN layer is applied independently to each position in the sequence, and the output is passed through normalization and residual connections, which help stabilize training and allow the model to learn better representations (Vaswani et al., 2017).

### Training and optimization

To train the transformer model, the parameters (weights) of the self-attention mechanism, the FFN, and the output layers are learned by minimizing a loss function (Song et al., 2016). In time series forecasting, the loss function is typically the mean squared error (MSE) between the predicted values and the true values (Jadon et al., 2024). The optimization process involves using gradient-based methods such as Stochastic Gradient Descent (SGD) or Adam to update the weights (Zhang et al., 2024). The gradient of the loss function with respect to the parameters is computed using backpropagation, and the parameters are updated iteratively to minimize the error.

### Applications of transformers in agriculture science

In agriculture, time series data is abundant and encompasses diverse types of information, such as weather conditions, crop yields, irrigation schedules, and pest outbreaks. There is a wide variety of research being done using machine learning and deep learning methods (Hill and Donald, 2003; Galford et al., 2008; Eerens et al., 2014; Zualkernan et al., 2023). Given the importance of time-dependent relationships in agricultural processes, transformers are well-suited to handle these complex datasets.

For instance, a transformer model can be used to forecast crop yields by learning patterns in historical weather data, soil moisture levels, and other environmental factors. The self-attention mechanism enables the model to focus on relevant time periods, such as those with specific weather patterns, which significantly affect crop growth. By capturing the complex dependencies in these time series, transformers can generate more accurate and robust predictions compared to traditional forecasting methods as observed in recent studies (Liu et al., 2022; Bi et al., 2023; Lu et al., 2024).

Furthermore, transformers can be used to optimize irrigation systems by predicting future water requirements based on historical weather patterns, soil moisture levels, and other factors (Deforce et al., 2024; Madhukumar et al., 2024). This can help farmers make more efficient use of water resources, leading to cost savings and improved sustainability.

In the agricultural sector, transformers offer the potential to revolutionize decision-making by providing more accurate forecasts for crop yields, livestock weight gains, and resource optimization. As transformers continue to evolve, their applications in time series analysis and agriculture are likely to expand, driving innovation in this critical field.

### Model evaluation

Regardless of the application for enhancing the productivity and efficiency of livestock systems, model evaluation is a critical component and should be ongoing as data are. After training a given model, the final model was used to predict the response variable for a given held-out samples. The squared difference between the predicted and the actual observed value can be calculated and divided by the number of rows in the held-out data set. This is the mean squared prediction error for the model:


$$\mathrm{MSPE}=\frac{\sum {\left(y_{i}(t)-{\hat{y}}_{i}(t)\right)}^{2}}{n}.$$

After training, the final model can be used to predict the full original data to calculate the MSE, MAE, $R^{2}$, ratio of performance deviation (RPD), and ratio of performance to inter-quartile (RPIQ). For MSE and MAE, the lower values shows a better performance, and for $R^{2}$, RPD, and RPIQ, higher values correspond to better models. Finally, the Gedeon method embedded in “H2O” is used to compute the importance of each predictor variable (Candel and LeDell, 2022). The variable importance determined by the deep learning algorithm are indicative of the most important variables at the time of the first layer.

Networks that are defined as feed-forward have a uni-directional information path (Goodfellow et al., 2016). The structure of the models is such that, any node information can only be sent to node $x_{i+1}$ (Dahikar and Rode, 2014). Backpropagation is used to fine-tune model weights, starting with the output layer and ending with the input. This process uses the difference between the true values of the data and the model output to determine how and what weights to modify (Dahikar and Rode, 2014). The loss function is specified as a measurement of the true value vs. the predicted value of the model. The error between the model and the true value of the test data is addressed in the regularization parameter, which specifically aims to minimize such error (Gu et al., 2018). Cross-validation provides additional metrics to look at how well the model is doing without needing to add more data. If the cross-validation metrics are not similar, the model may not be doing well.

## Conclusion

Capturing the dependency in time series data is crucial for making inferences and predictions, as it helps uncover underlying patterns that can drive insights. Dynamical models are powerful tools to properly assess the behavior of environmental and biological phenomena, as they allow for the representation of time-dependent processes and interactions between variables. These models, often based on differential equations or system dynamics, offer a structured approach to understanding complex systems.

Deep Neural Networks, on the other hand, excel at learning intricate, non-linear relationships within the data, making them particularly effective for capturing complex patterns that may be difficult to model explicitly. Deep Neural Networks can adapt to a wide range of inputs, such as unstructured data like images, audio, or text, and structured data like sensor readings or historical time series datasets like satellite imagery.

However, the quality of the data and the appropriate choice of DNN structure are paramount in ensuring the success of deep learning models. High-quality data, with accurate and relevant features, is essential for the model to learn meaningful representations. In addition, selecting the right architecture, including the number of layers, types of neurons, and activation functions, is critical in achieving optimal performance. Overfitting and underfitting are common challenges that arise from poor model choices, and they can be mitigated by techniques such as regularization, cross-validation, and careful data preprocessing.

Moreover, the integration of domain-specific knowledge, whether through the inclusion of expert-designed features or the use of hybrid models that combine traditional dynamical models with machine learning approaches, can enhance the ability of DNNs to model environmental phenomena more effectively. Thus, achieving accurate predictions requires not only sophisticated algorithms but also a deep understanding of the system under study and a strong foundation in data science.

## Data Availability

Data sharing is not applicable to this article as no datasets were generated or analyzed during the current study.

## Abbreviations:

ARIMAX: AutoRegressive Integrated Moving Average with eXogenous inputs;
DNN: deep neural network;
RNN: recurrent neural network;
MSE: mean squared error;
MSPE: mean squared prediction error;
AIC: Akaike information criterion;
BIC: Bayesian information criteria
